# Detection of Nonexudative Macular Neovascularization on Structural OCT Images Using Vision Transformers

**DOI:** 10.1016/j.xops.2022.100197

**Published:** 2022-07-08

**Authors:** Yuka Kihara, Mengxi Shen, Yingying Shi, Xiaoshuang Jiang, Liang Wang, Rita Laiginhas, Cancan Lyu, Jin Yang, Jeremy Liu, Rosalyn Morin, Randy Lu, Hironobu Fujiyoshi, William J. Feuer, Giovanni Gregori, Philip J. Rosenfeld, Aaron Y. Lee

**Affiliations:** 1 University of Washington, Department of Ophthalmology, Seattle, Washington; 2 Department of Ophthalmology, Bascom Palmer Eye Institute, University of Miami Miller School of Medicine, Miami, Florida; 3 Department of Robotic Science and Technology, Chubu University, Aichi, Japan

**Keywords:** Age-related macular degeneration, Deep learning, Macular neovascularization, OCT, AI, artificial intelligence, AMD, age-related macular degeneration, CNN, convolutional neural network, DLS, double-layer sign, GA, geographic atrophy, iAMD, intermediate age-related macular degeneration, IoU, Intersection over Union, MNV, macular neovascularization, neMNV, nonexudative macular neovascularization, NPV, negative predictive value, OCTA, OCT angiography, PPV, positive predictive value, ROC, receiver operating characteristic, RPE, retinal pigment epithelium, SS-OCT, swept-source OCT, SS-OCTA, swept-source OCT angiography, ViT, Vision Transformer

## Abstract

**Purpose:**

A deep learning model was developed to detect nonexudative macular neovascularization (neMNV) using OCT B-scans.

**Design:**

Retrospective review of a prospective, observational study.

**Participants:**

Normal control eyes and patients with age-related macular degeneration (AMD) with and without neMNV.

**Methods:**

Swept-source OCT angiography (SS-OCTA) imaging (PLEX Elite 9000, Carl Zeiss Meditec, Inc) was performed using the 6 × 6-mm scan pattern. Individual B-scans were annotated to distinguish between drusen and the double-layer sign (DLS) associated with the neMNV. The machine learning model was tested on a dataset graded by humans, and model performance was compared with the human graders.

**Main Outcome Measures:**

Intersection over Union (IoU) score was measured to evaluate segmentation network performance. Area under the receiver operating characteristic curve values, sensitivity, specificity, and positive predictive value (PPV) and negative predictive value (NPV) were measured to assess the performance of the final classification performance. Chance-corrected agreement between the algorithm and the human grader determinations was measured with Cohen’s kappa.

**Results:**

A total of 251 eyes from 210 patients, including 182 eyes with DLS and 115 eyes with drusen, were used for model training. Of 125 500 B-scans, 6879 B-scans were manually annotated. A vision transformer segmentation model was built to extract DLS and drusen from B-scans. The extracted prediction masks from all B-scans in a volume were projected to an en face image, and an eye-level projection map was obtained for each eye. A binary classification algorithm was established to identify eyes with neMNV from the projection map. The algorithm achieved 82%, 90%, 79%, and 91% sensitivity, specificity, PPV, and NPV, respectively, on a separate test set of 100 eyes that were evaluated by human graders in a previous study. The area under the curve value was calculated as 0.91 (95% confidence interval, 0.85–0.98). The results of the algorithm showed excellent agreement with the senior human grader (kappa = 0.83, *P <* 0.001) and moderate agreement with the junior grader consensus (kappa = 0.54, *P <* 0.001).

**Conclusions:**

Our network (code is available at https://github.com/uw-biomedical-ml/double_layer_vit) was able to detect the presence of neMNV from structural B-scans alone by applying a purely transformer-based model.

Type 1 macular neovascularization (MNV) is the most common form of MNV seen in eyes with age-related macular degeneration (AMD).^1^^,^^2^ This neovascularization arises from the choroid and grows under the retinal pigment epithelium (RPE) and resides between Bruch’s membrane and the RPE.^3^ In AMD, these nonexudative neovascular lesions have an increased risk of progressing to exudation and vision loss.^4^, ^5^, ^6^ Because early detection and treatment of exudative AMD have been shown to result in better visual acuity outcomes, it is important to identify and closely follow nonexudative MNV (neMNV) even before exudation develops so that treatment can be initiated once symptomatic exudation arises. OCT angiography (OCTA) is the noninvasive imaging strategy of choice for the detection of neMNV.^7^^,^^8^

Although OCTA is able to detect neMNV, not all clinical practices are equipped with an OCT instrument capable of angiographic imaging. However, most practices are equipped with standard OCT instruments that provide structural B-scan images. The presence of type 1 neMNV has been associated with the presence of a double-layer sign (DLS), also known as a “shallow irregular RPE elevation,” on structural OCT B-scan images.^9^^,^^10^ A cost-effective strategy to detect these nonexudative neovascular lesions without requiring the use of the more expensive OCTA technology would be to train clinicians to detect neMNV using structural OCT B-scans.

Shi et al^9^ investigated whether graders could accurately identify the presence of a DLS in eyes with type 1 neMNV. After training on eyes with known subclinical neovascular lesions, the graders assessed a total of 100 eyes with AMD in which 20 eyes had both drusen and type 1 neMNV, 13 eyes had geographic atrophy (GA) along with type 1 neMNV, 44 eyes had only drusen, and 23 eyes had only GA. Although a statistically significant association was found between the presence of the DLS and type 1 neMNV, the sensitivity, specificity, positive predictive value (PPV), and negative predictive value (NPV) for the junior graders were 73%, 84%, 69%, and 86%, respectively, and the sensitivity, specificity, PPV, and NPV for the senior grader were 88%, 87%, 76%, and 94%, respectively. These results suggested that training and experience should yield improved results, and the grading of these structural OCT images for the presence of a DLS could be approached by developing machine learning algorithms to detect these lesions.

In this study, we aimed to develop a deep learning algorithm to detect a DLS based on cross-sectional structural OCT B-scans. Vision Transformer (ViT)^11^ is now considered the state of the art in many computer vision tasks. We trained a ViT segmentation model using eyes with and without type 1 neMNV that was confirmed on swept-source OCTA (SS-OCTA) imaging. A large dataset of annotated structural B scans was fed into the algorithm to train the model. After training and preliminary testing, the machine learning model was finally applied on the same dataset of 100 eyes that were evaluated by human graders in our previous study.^9^ The sensitivity, specificity, NPV, and PPV of the machine learning algorithm were then compared with the performance of the human graders.

## Methods

Patients with AMD were enrolled in a prospective OCT imaging study at the Bascom Palmer Eye Institute. The Institutional Review Board of the University of Miami Miller School of Medicine approved this study. Informed consent was obtained from all patients. The study was performed in accordance with the tenets of the Declaration of Helsinki and complied with the Health Insurance Portability and Accountability Act of 1996.

Swept-source OCTA (PLEX Elite 9000; Carl Zeiss Meditec, Inc) images were acquired using a 6 × 6-mm scan pattern centered on the fovea. The SS-OCT laser operates at a central wavelength of 1050 nm at a speed of 100 000 A-scans per second. At the level of the retina, the full width at half-maximum axial resolution is approximately 5 μm, with an estimated lateral resolution at the retinal surface of appropriately 20 μm. In the 6 × 6-mm scan pattern, a single B-scan consists of 500 A-scans, and 2 B-scans are repeated at each of 500 B-scan positions. En face flow images were generated by the instrument using the OCT microangiography algorithm, as previously described.^11^ For the visualization of the neMNV, a custom segmentation strategy was used to create slabs extending from the RPE to RPE-fit. This feature is available on the commercial instrument and allows the reviewer to select prespecified boundary layers, and in this study, the boundary layers selected for segmentation were the RPE and the RPE-fit, also known as “Bruch’s membrane.” Obvious artifacts in the boundaries generated by the automated segmentations were corrected using the editing tool.

All AMD eyes included in this study showed no evidence of exudation based on the absence of macular fluid after a review of the retina thickness maps and B-scans from the SS-OCT images. Nonexudative AMD eyes were classified by clinical examinations as intermediate AMD (iAMD) or late AMD. Intermediate AMD was defined as eyes with drusen of ≥ 125 mm in diameter or pigmentary changes, but without evidence of GA or exudation. Pigmentary changes were defined as any foci of hyperreflectivity within the retina or foci of thickened RPE identified on B-scan and causing a choroidal hypotransmission defect that were associated with medium (> 63 μm and ≤ 125 μm) or large (> 125 μm) drusen.^12^, ^13^, ^14^ Late nonexudative AMD was defined by the presence of GA, also known as “complete RPE and outer retinal atrophy,”^15^^,^^16^ in the absence of exudation. The diagnosis of neMNV was based on SS-OCTA imaging as previously described.^6^^,^^7^ Eyes with neMNV included eyes with both iAMD and late AMD with GA. Eyes without neMNV were chosen on the basis of the presence of iAMD or late nonexudative AMD, and their clinical characteristics were otherwise similar to the eyes with neMNV.

**Figure 1:**
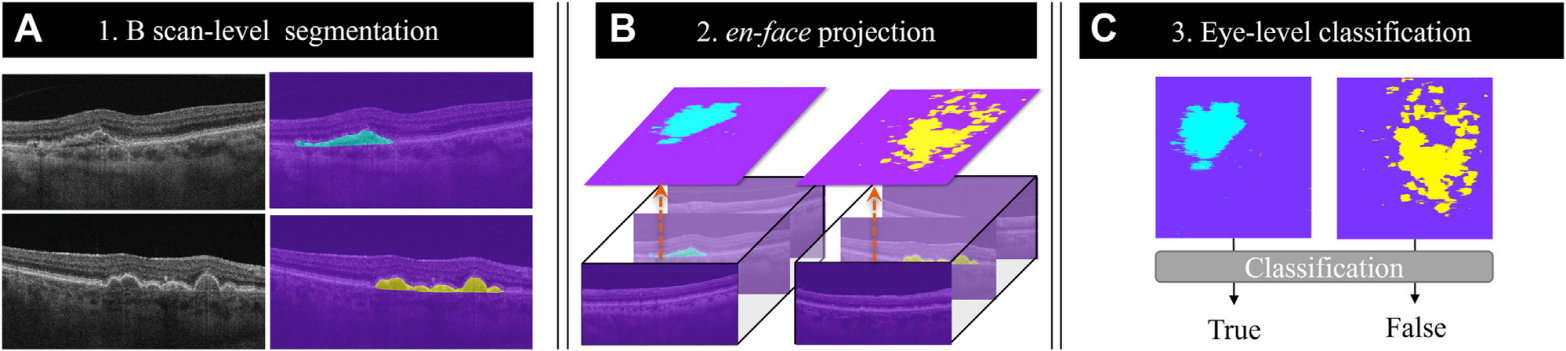
Process overview. **A,** Double-layer signs (DLSs) and drusen were extracted from B-scans by a segmentation model. The detected DLSs and drusen are depicted in **blue** and **yellow**, respectively. **B,** After processing predictions for all B-scans, we generated en face maps. **C,** Eyes with macular neovascularization (MNV) were then identified on the basis of the en face projection map.

B-scans were annotated by graders (M.S., Y.S., X.J., L.W., R.L., C.L., J.Y., J.L., R.M.) from Bascom Palmer Eye Institute using Photoshop (Adobe Creative Cloud) based on the presence of neMNV and drusen, as defined by SS-OCTA imaging. Only drusen > 63 μm in diameter were annotated on B-scans. Consensus gradings of the pathologies were performed by 2 graders with a senior grader (P.J.R.) adjudicating any disagreements. Pathologies were confirmed on SS-OCTA. All the annotated B-scans along with SS-OCT data were then sent to the University of Washington for algorithm training. The University of Washington team built a framework that first extracts DLS and drusen for each B scan using a segmentation model, and then classifies eyes with MNV (Fig 1).

### Test Set Description

A completely separate set of patients were used as a test set; these patients were not used for training and internal validation. The test set consisted of 100 AMD eyes with drusen and GA, with or without nonexudative type 1 MNV; this is the same test set that was used by human graders in our previous study. This test set is shown in Table 1.^9^ The consensus grading results of these 100 eyes were used as the ground truth to be compared with the output from the current model. Sensitivity, specificity, PPV, and NPV of the artificial intelligence (AI) algorithm for identifying DLS associated with neMNV were calculated.

**Table 1:** Presence of Subclinical MNV in Test Eyes with Nonexudative AMD

Group	Intermediate AMD	Late AMD
	(n = 64)	(n = 36)
Presence of subclinical MNV (n = 33)	20	13
Absence of subclinical MNV (n = 67)	44	23

AMD = age-related macular degeneration; MNV = macular neovascularization.

### ViT-Based Segmentation Model

**Figure 2:**
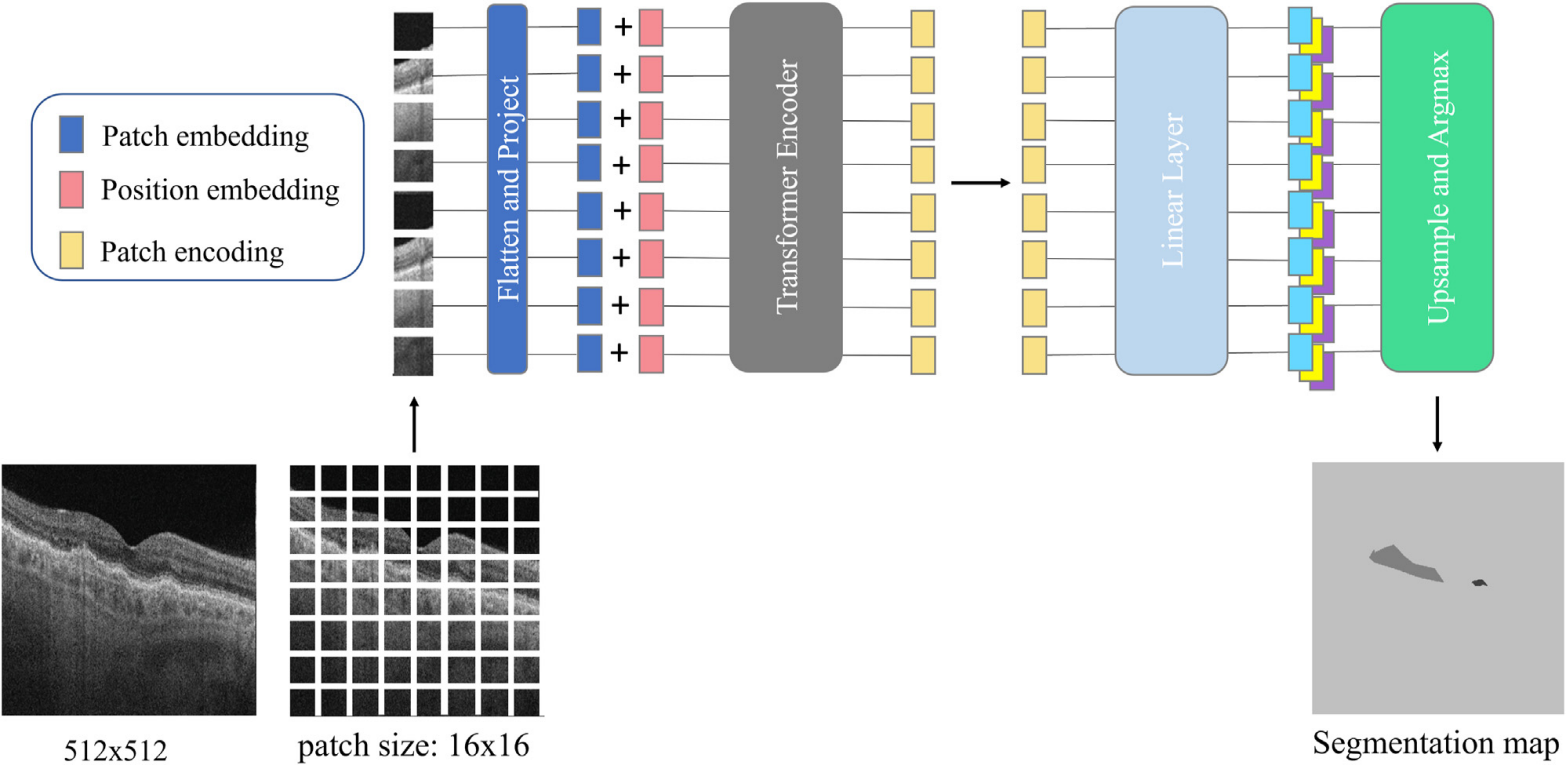
Framework of Vision Transformer segmentation model; 512 × 512 images were used as input; and 16 × 16 image patches were projected to a sequence of embeddings and then encoded with a transformer and reshaped into a segmentation map.

The segmentation model was built using a fully ViT-based encoder-decoder architecture^17^ mapping a sequence of patch embeddings to pixel-level class labels. An overview of the model is shown in Figure 2. Because ViT is relatively novel and has limited application in ophthalmology, we briefly describe the architecture of our model. First, an input image was split into a sequence of patches. Each patch was flattened into a 1-dimensional vector and then fed into a linear projection layer that would produce a sequence of patch embeddings. To retain positional information, learnable position embeddings are added to the patch embeddings to get the resulting input sequence of tokens. A transformer layer consists of a multi-headed self-attention block followed by a point-wise multi-layer perceptrons block of 2 layers with layer norm applied before every block and residual connections added after every block. The transformer encoder was applied to the sequence of tokens to generate a contextualized encoded sequence. The decoder learns to map patch-level encodings coming from the encoder to patch-level class labels. A pointwise linear layer was applied to the patch-level encodings to produce patch-level class logits. The sequence was then reshaped into a 2-dimensional feature map and upsampled using bilinear interpolation to the original image size. A softmax was then applied on the class dimension to obtain the final segmentation map.

The resolution of the original B-scan image was 500 pixels in width and 1536 pixels in height. The B-scan was then systematically cropped into 500 × 768 so that the retinal layer comes to the center of the cropped image. During the training, we applied mean subtraction, random resizing of the image to a ratio between 0.5 and 2.0, and random left-right flipping for data augmentation purposes. We randomly cropped large images and pad small images to a fixed input size of 512 × 512. The original B-scan image was 1-channel grayscale, but we artificially created a 3-channel grayscale image in RGB to match the dimension of ViT pretrained model by simply duplicating the grayscale image 3 times and stacking them in the third dimension. The input images were then normalized across the RGB channels with mean (0.5, 0.5, 0.5) and standard deviation (0.5, 0.5, 0.5). The output of the network was the same size as the input but had 3 channels corresponding to the background, DLS, and drusen. Our model was trained end-to-end with a per-pixel cross-entropy loss with stochastic gradient descent optimizer with the base learning rate of 1 × 10^−3^ and polynomial decay scheduler with the power of 0.9, following fine-tuning procedure in the previous study.^17^ We trained 64 epochs with a batch size of 8. At inference time, argmax was applied after upsampling to obtain a single class label per pixel.

For the semantic segmentation task at higher resolution (512 × 512), we fine-tuned the model, keeping patch size fixed and bilinearly interpolated the pretrained position embeddings according to their original position in the image to match the fine-tuning sequence length, following ViT.^11^ Our backbone ViT model had 12 layers, 768 token sizes, and 12 heads, and was pretrained on ImageNet-21k at image resolution 224 × 224, which was publicly available provided by the image classification library timm.^18^

As a point of comparison, we also trained a U-Net^19^ model using a traditional fully convolutional approach on the same dataset as our ViT segmentation model. Just as in ViT, systematically cropped 500 × 768 B scans were used. The images were then rescaled to 512 × 512 and normalized to a range between 0 and 1. The model was trained with a cross-entropy loss function using Adam optimizer. We chose a batch size of 8. The learning rate was initially set to 1 × 10^−4^, and decay over each update was set to the initial learning ratio divided by epochs. Basic data augmentations such as shift, flip, and rotation were applied. Furthermore, Spatial Dropout that drops entire feature maps instead of individual elements was additionally applied to regularize the activations and reduce overfitting.

### Classification Criteria

**Figure 3:**
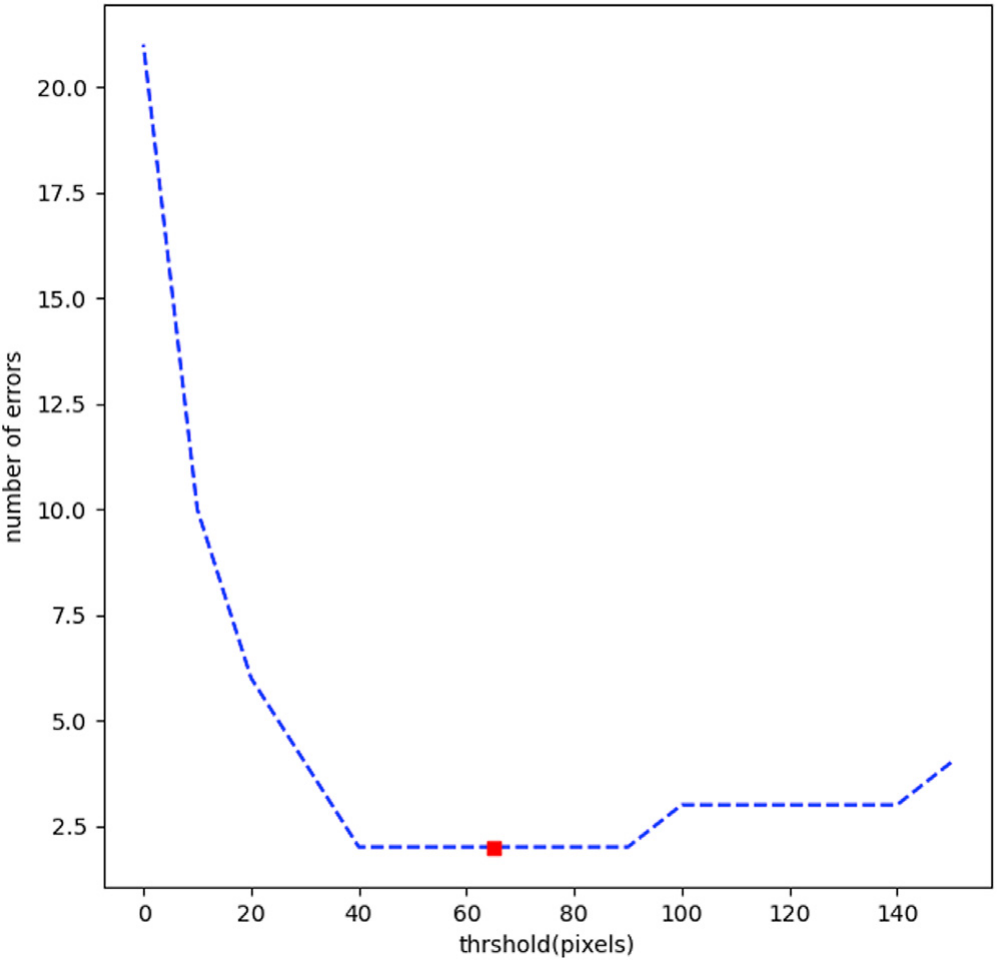
Threshold versus Classification errors. On each 128 × 128 en face projection map on the validation set, we observed how the number of classification errors changed with regard to the threshold. Between threshold values were 40 to 60, the errors were minimum, and 65 was the middle point.

We built a binary classification algorithm that identifies eyes with MNV from an en face prediction map generated by processing all 500 slices of B-scans and calculating prediction masks using the segmentation model as shown in Figure 1. The en face images had 500 (width) × 500 (height) that corresponded to the original width and depth of the B-scans, respectively. From the en face images, we first rescaled them to 128 × 128 to reduce noise and then extracted all connected blobs of DLS lesions. The size of the largest component for each eye was measured, and then DLS labels were assigned to the eye if the largest component size was > 65 pixels. To set the threshold value, we processed the described criteria to an internal validation set and observed how the number of classification errors changed. We then selected the center point of component size with the lowest error as our threshold (Fig 3).

### Assessment of DLS Determinations

The performance of DLS determinations by the algorithm and human graders was summarized using sensitivity, specificity, and PPV and NPV. Chance-corrected agreement between the algorithm and human grader determinations was summarized with Cohen’s kappa (kappa < 0.4: poor; kappa 0.4–0.75: fair to good; kappa > 0.75; excellent).^20^

## Results

**Figure 4:**
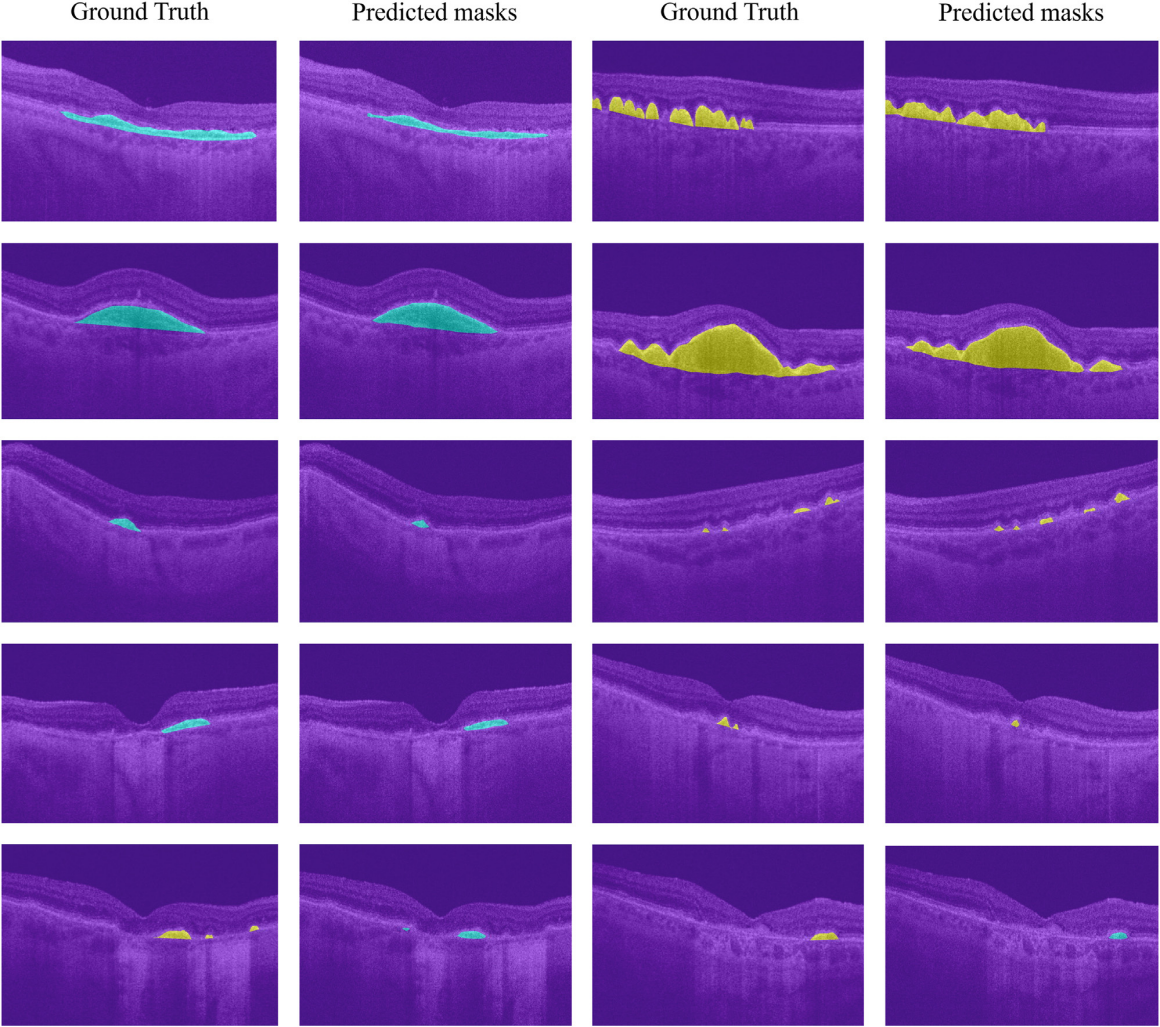
Prediction examples from the validation set. Grounded Truth **(left)** and predicted masks **(right)** overlaid on input B scan. **First to fourth rows:** The detected double-layer signs (DLSs) and drusen are depicted in **blue** and **yellow**, respectively. Our segmentation model was mostly able to properly extract DLSs and drusen at different locations/volumes. **Fifth row:** Failure case examples. Our segmentation model tends to mislabel drusen to DLSs on eyes with geographic atrophy.

A total of 251 eyes from 210 AMD patients were imaged using SS-OCTA and included in this study, including 182 eyes with a DLS and 115 eyes with drusen. The data were partitioned into 70% for training and 30% for validation sets at the patient level. Of a total of 125 500 B-scans, 5256 B-scans and 1623 B-scans were used for manual labeling of training and validation, respectively, and provided for segmentation model training. Mean Intersection over Union (IoU) was DLS: 58.80%, drusen: 61.11% for the Transformer model, and DLS: 55.39%, drusen: 54.30% for the U-Net model on the validation model. Some examples of predicted masks are shown in Figure 4.

**Figure 5:**
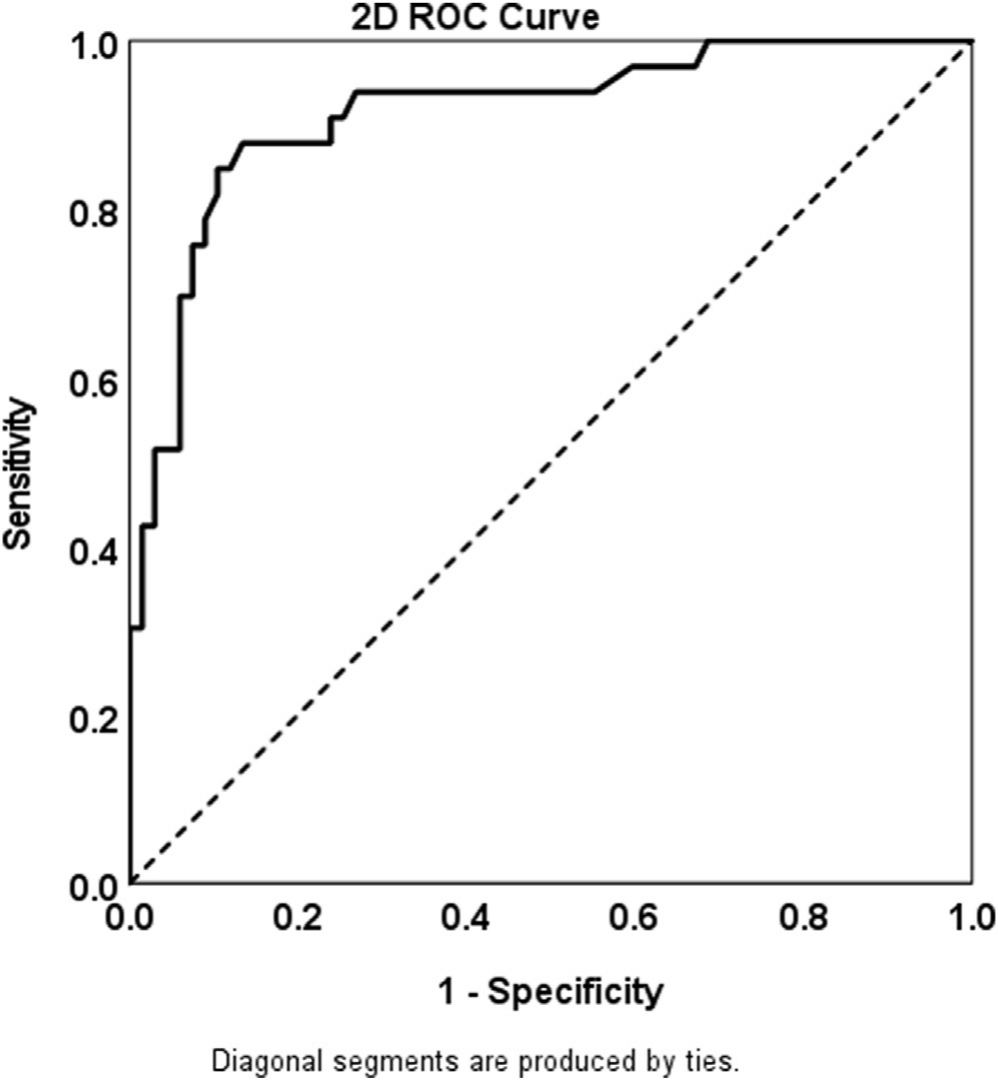
Receiver operating characteristic (ROC) curve. The ROC for prediction of eyes with macular neovascularization based on the predicted double-layer signs lesion size 2-dimensionally (2D) measured from en face projection map. Area under the curve = 0.91 (95% confidence interval, 0.85–0.98).

A separate test set, which was held out from training and validation, consisted of 100 eyes with nonexudative AMD from 94 patients (Table 1). The test set contained 64 eyes with iAMD, which included 33 eyes with neMNV, of which 20 eyes had iAMD and 13 eyes had GA, and 67 eyes without neMNV, of which 44 had iAMD and 23 had GA. All the results are summarized in Table 2. Our model, referred to as the “AI grader,” detected DLSs in 27 of 33 eyes with subclinical MNV and did not detect a DLS in 60 of 67 eyes without MNV. The sensitivity, specificity, PPV, and NPV results of the model were 82%, 90%, 79%, and 91%, respectively. The receiver operating characteristic (ROC) analysis (Fig 5) found that the DLS area size extracted from our ViT model was effective in predicting the presence of DLS. According to ROC analysis, the area under the curve value was calculated as 0.91 (95% confidence interval, 0.85–0.98).

**Table 2:** Sensitivity, Specificity, and Predictive Values of Double-Layer Sign for Identifying Subclinical Macular Neovascularization in Nonexudative AMD

Values	AMD (n = 100)			Intermediate AMD (n = 64)			Late AMD (n = 36)		
	*AI Grader*	*Consensus from Junior Grader*	*Results from Senior Grader*	*AI Grader*	*Consensus from Junior Grader*	*Results from Senior Grader*	*AI Grader*	*Consensus from Junior Grader*	*Results from Senior Grader*
Sensitivity	0.82	0.73	**0.88**	0.75	0.85	**0.90**	**0.92**	0.54	0.85
Specificity	**0.90**	0.84	0.87	**0.96**	0.84	0.93	**0.78**	0.83	0.74
PPV	**0.79**	0.69	0.76	**0.88**	0.71	0.86	**0.71**	0.64	0.65
NPV	0.91	0.86	**0.94**	0.89	0.93	**0.95**	**0.95**	0.76	0.89
*P* value	≤0.001	≤0.001	≤0.001	≤0.001	≤0.001	≤0.001	≤0.001	0.056	≤0.001

AI = artificial intelligence; AMD = age-related macular degeneration; NPV = negative predictive value; PPV = positive predictive value.

### Comparison with Human Graders

To test the performance of our current model, the results from the model were compared against human junior graders and a senior grader using the same 100 eyes from our previous study.^9^ As previously described, 2 junior graders read the scans from 100 eyes separately and then reached a consensus grading. They detected DLSs in 24 of 33 eyes with neMNV and did not detect DLSs in 56 of 67 eyes without MNV. Their sensitivity, specificity, PPV, and NPV were 73%, 84%, 69%, and 86%, respectively. The senior grader detected DLSs in 29 of 33 eyes with neMNV and did not detect DLSs in 58 of 67 eyes without MNV, achieving a sensitivity, specificity, PPV, and NPV of 88%, 87%, 76%, and 94%, respectively. For all graders, there were statistically significant associations between type 1 MNV and the presence of the DLS (*P* ≤ 0.001). Compared with human graders, the AI grader consistently performed better than junior graders and as well as the senior grader. Above all, the AI grader showed robust performance on eyes with late AMD, whereas human graders had more difficulty in identifying DLSs in eyes with GA. Agreement between the AI and the senior grader was excellent (kappa = 0.83, *P <* 0.001), whereas agreement with the junior grader consensus was moderate (kappa = 0.54, *P <* 0.001). Agreement between junior and senior human graders was good (kappa = 0.68, *P <* 0.001). Our code is available at https://github.com/uw-biomedical-ml/double_layer_vit.

## Discussion

Previous studies^9^^,^^10^ have shown that the presence of DLSs on structural OCT images could be used to predict the presence of neMNV. We previously found that human graders, especially experienced graders, were able to identify the MNV based on the presence of DLS seen on structural B scans with high accuracy. In this study, we developed a machine learning framework to identify DLS associated with neMNV. We used a ViT segmentation model to detect DLS from structural OCT B-scan. Eyes with neMNV were then identified by aggregating these detected lesions on eye level. The presented algorithm showed similar ability compared with experienced human graders on this task.

The ViT showed significantly better performance than the U-Net model on the segmentation task. Although U-Net is just one example of the convolutional neural network (CNN) and ViT is just one example of Transformer models, the performance gap could have arisen from the fundamental difference in their model architectures. The ViT is different from CNN. Convolutional neural networks start with a feature of large spatial sizes and a small channel size and gradually increase the channel size while decreasing the spatial size. In ViT, input images are divided into 16 × 16 patches and fed to the transformer network; except for the first embedding layer, there is no convolution operation in ViT, and the position interactions occur through the self-attention layers. Although CNNs have restricted spatial interactions, ViT uses multi-head self-attention that allows all the positions in an image to interact. These characteristics of ViT might have helped with improving segmentation performance, especially in eyes with GA.

### Study Limitations

Because our model was tested only on structural SS-OCT scans with AMD, the model should be applied only to SS-OCT scans with AMD. Our model also has some limitations in detecting small lesions. Figure 5 shows the ROC curve that describes classification performance based on the area size of the extracted largest DLS lesion per eye. There is a strong correlation between lesion size and its performance. In general, small lesions are difficult to extract by our model and difficult to distinguish between DLSs or drusen. The performance may improve if we add additional examples to the training dataset. Another approach is to add lesion size reweighting to our loss function during the ViT segmentation model training.

## Conclusions

The presented frameworks using ViT predicted the presence of DLSs from the SS-OCT scans. The evaluation demonstrated that segmentation performance is consistent and reliable, and can substantially improve sensitivity and specificity in the final classification task. The development of a machine-learning algorithm to identify subclinical MNV based on spectral-domain OCT imaging is under way.
